# Potential Antimicrobe Producer of Endophytic Bacteria from Yellow Root Plant (*Arcangelisia flava* (L.)) Originated from Enggano Island

**DOI:** 10.1155/2022/6435202

**Published:** 2022-11-16

**Authors:** Sipriyadi, Masrukhin, Risky Hadi Wibowo, Welly Darwis, Salprima Yudha, Ismu Purnaningsih, Resli Siboro

**Affiliations:** ^1^Faculty of Mathematics and Natural Science, University of Bengkulu, Jl. W.R Supratman, Kandang Limun, Bengkulu 38122, Indonesia; ^2^Master Program of Biology, Faculty of Mathematics and Natural Science, University of Bengkulu, Jl. W.R Supratman, Kandang Limun, Bengkulu 38122, Indonesia; ^**3**^ Research Center for Biosystematics and Evolution, National Research and Innovation Agency (BRIN), Cibinong, Bogor 16911, West Java, Indonesia; ^**4**^ Chemistry Department, Faculty of Mathematics and Natural Science, University of Bengkulu, Jl. W.R Supratman, Kandang Limun, Bengkulu 38122, Indonesia; ^**5**^ Research Center of Natural Products and Functional Materials, University of Bengkulu, J.W.R. Supratman, Kandang Limun, Bengkulu 38122, Indonesia; ^**6**^ Directorate for Scientific Collection, National Research and Innovation Agency (BRIN), Cibinong, Bogor 16911, West Java, Indonesia

## Abstract

Exploration studies of endophytic bacteria from *Arcangelisia flava* (L.) and their potential have not much been conducted. This research aims to explore and characterize the antimicrobial activity of endophytic bacteria in *A. flava* against pathogenic bacteria. This research consists of several steps including the isolation of bacteria, screening of the antimicrobial activity assay using the dual cross streak method, molecular identification through 16s rDNA analysis, and characterization of bioactive compound production through PKS-NRPS gene detection and GC-MS analysis. There are 29 endophytic bacteria that were successfully isolated from *A. flava*. The antimicrobial activity showed that there are four potential isolates AKEBG21, AKEBG23, AKEBG25, and AKEBG28 that can inhibit the growth of pathogenic bacteria such as *Escherichia coli, Staphylococcus aureus,* and *Pseudomonas aeruginosa*. The 16S rDNA sequence analysis showed that these isolates are identified as *Bacillus cereus*. These four isolates are identified as able to produce the bioactive compounds through the detection of polyketide synthase (PKS) and nonribosomal peptide synthase (NRPS)-encoding genes. *B. cereus* AKEBG23 has the highest inhibition against pathogenic bacteria, and according to the GC-MS analysis, five major compounds are allegedly involved in its antimicrobial activity such as butylated hydroxytoluene (BHT), diisooctyl phthalate, E-15-heptadecenal, 1-heneicosanol, and E-14-hexadecenal. This result suggested that *B. cereus* AKEBG23 as the endophytic bacterium from *A. flava* has a beneficial role as well as the plant itself. The bacterium produces several bioactive compounds that are allegedly involved in its antimicrobial activity against pathogenic bacteria.

## 1. Introduction

**Figure 1:**
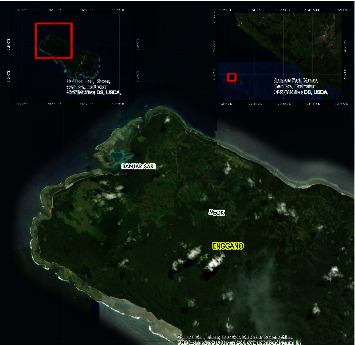
Sampling area of A. flava (L.) samples in Enggano Island (map sources: google earth, Bengkulu provincial administration map).

Enggano Island is one of the outer islands in Indonesia which administratively belongs to the Bengkulu Province (Figure 1). As one of the outer islands, Enggano has a high floral diversity in several ecosystems such as mangrove, beach, riparian, natural forest, and swamp ecosystems. The high floral diversity in Enggano included the medicinal plants utilized by local people as the traditional medicine inherited from the former generation [1]. *Arcangelisia flava* (L.) is one of the medicinal plants found in Enggano. The local people named “kayu kuning” (yellow wood) or “akar kuning” (yellow root), which generally consists of three different species such as *Arcangelisia flava* (L.), *Fibraurea tinctoria* and *Coscinium fenestratum* [2]. *A. flava* is widely distributed in Indonesia, such as in Sumatera, Kalimantan, Sulawesi, Java, and Maluku [3].

The local people use the *A. flava* plants to treat several diseases such as malaria, dysentery, and fever [4, 5]. Previous research has also shown the potency of *A. flava* as the producer of an antioxidant, antidiabetic, and antimicrobial compound such as berberine [6–8]. Previous research also reported that the alkaloid extract from *A. flava*. has the potential to treat skin-related fungal infections caused by *Candida albicans* and *Trichophyton mentagrophytes* [6].

The potential of *A. flava* as a medicinal plant is not only found in the direct usage of the plant metabolite, but also its endophytic microbes reside in it. Several endophytic microbes isolated from *A. flava* leaves produce the antimicrobial compound. Previous research reported that *coelomycetes* AFKR-18, the endophytic fungi isolated from *A. flava,* produce pachybasin as the major antimicrobial and antifungal compound against *Escherichia coli, Bacillus subtilis, Micrococcus luteus, Candida albicans, and Aspergillus flavus* [9]. *Coelomycetes* also produce phloroglucinol which has strong antimicrobial activity against *E. coli* [10]. Instead of fungi, a recent study also reported that some endophytic bacteria from *A. flava* have antimicrobial activity, such as *Bacillus cereus* AKEBG28, which has antimicrobial activity against *Escherichia coli* and *Staphylococcus aureus* [11].

According to these research works, the endophytic microbe of *A. flava* has the potential as the antimicrobial compound producer. The data of endophytic microbes from *A. flava* from Enggano Island are not well studied, especially related to the beneficial compound produced by the microbes. Therefore, the objectives of this research are to characterize the endophytic bacteria isolated from *A. flava* originated from Enggano Island as the potential antimicrobe and conduct the profiling of the bioactive compound produced by the bacteria.

## 2. Materials and Methods

### 2.1. Sample Collection

The *A. flava* samples were collected from Enggano Island (Figure 1). The plant was collected and divided into 3 parts that are roots, stems, and leaves, and each sample was stored and will be used as a source for microbial isolation.

### 2.2. Isolation and Purification of Bacteria

Isolation of endophytic bacteria was conducted using the serial dilution method from 10^−1^ to 10^−8^. The plant tissue was surface-sterilized, crushed, and diluted in a sterile saline solution. As much as 0.1 mL of each dilution was inoculated to nutrient agar (NA) medium using the spread plate method and incubated at 30°C for 24–48 hr. The grown colony was then gradually purified by inoculating to the new NA medium.

### 2.3. Antimicrobial Assay

Screening of potential antimicrobial isolates was performed using the cross streak method [12] with a few modifications against four pathogenic microbes, namely, *Staphylococcus aureus, Escherichia coli, Pseudomonas aeruginosa, and Candida albicans.* The isolate was cultured overnight in tryptic soy broth (TSB) and adjusted using the McFarland turbidity standard of 0.5 so that the cells reach 1.5 × 10^8^ CFU. As much as 1% (v/v) of pathogenic microbes were inoculated into 100 mL of tryptic soy agar (TSA) at ±40°C, homogenated, and poured onto a sterile Petri dish. The endophytic bacteria were then streaked in the TSA medium containing pathogenic microbes and incubated at 30°C for 48 hr. The clear zone around the potential endophytic bacteria indicates the antimicrobial activity. The diameter of the zone was measured to determine the strength of the antimicrobial nature of each isolate. The further antimicrobial assay was performed for the selected potential isolates with a similar method with three replicates. The assay was carried out by using a pure bacterial colony, filtrate of the medium, cell pellets, and ethyl acetate extract. As for the filtrate and ethyl acetate extract, the antimicrobial assay used was the disk diffusion method [13, 14].

### 2.4. Identification of Bacteria

The sequencing of 16S rDNA was used to identify the endophytic bacteria. Several selected isolates were grown in NA for 48 h for DNA extraction. The DNA was extracted using a Geneaid Presto bacterial DNA Extraction Kit (Geneaid Biotech Ltd.; New Taipei City, TW) according to the standard protocol provided by the manufacturer. Pure genomic DNA was then amplified for 16S rDNA using universal primer 63F/1387R [15] using the T100 Thermal Cycler (Bio-Rad Laboratories; Hercules, CA, USA). The PCR product was then sequenced using the Sanger sequencing method outsourced in a service laboratory. The 16S rDNA sequences were analyzed using ChromasPro software (Version 1.7.7; Technelysium Pte Ltd.; South Brisbane, QLD, AU) for the quality checking and trimming process. The assembled forward and reverse 16s rDNA sequences were compared to an online database in GeneBank through the basic local alignment tool for nucleotide (BLAST-N) [16]. The neighbor-joining tree was generated using MEGA version 7.0 [17] using the bootstrap method, with 1000x replicates.

### 2.5. Extraction and Profiling of the Bioactive Compound

Profiling of bioactive compounds was conducted through ethyl acetate extraction and then continued in gas chromatography-mass spectrophotometry (GC-MS) analysis. As much as 10 mL of 24 h potential bacterial isolates were inoculated into 1 L of the TSB medium, the suspension was incubated in a shaker incubator at 30°C for 72 h. The extraction of the metabolite from the potential isolates was conducted by the addition of ethyl acetate 1 : 1 (v/v) and shaking for 20 min. The upper layer of the suspension (solvent layer) was then separated and evaporated in a rotary evaporator at 42°C. The evaporated extract obtained then weighted and diluted in 1 mL dimethyl sulfoxide 50% (DMSO) to determine the concentration. GC-MS analysis was performed using Shimadzu GC-MS-QP 2010 (Shimadzu Corporation; Tokyo, JPN) with RTX-5MS column. The result of mass spectra was then compared to the National Institute of Standards and Technology (NIST) database version 11 (NIST; Gaithersburg, MD, USA).

### 2.6. Amplification of PKS and NRPS-Encoding Gene

Amplification of polyketide synthase (PKS) and nonribosomal peptide synthetase (NRPS)-encoding genes was performed on the selected potential isolates with the highest antimicrobial activity. The PKS-encoding gene was amplified using a specific primer for the ketosynthase (KS) domain, degKS2F.gc 5′- GCSATGGAYCCSCARCARCGSVT-3′ and the reverse primer, deg.KSR5.gc 5′-GTSCCSGTSCCRTGSSCYTCSAC-3′ while NRPS-encoding gene was amplified using domain NRPS specific primer forward deg.NRPS-1F.i 5′-AARDSIGGIGSIGSITAYBICC-3 and reverse deg.NRPS-4R.i 5′-CKRWAICCICKIAIYTTIAYYTG-3′ [18]. PCR amplification was conducted using the T100 Thermal Cycler with the following PCR mix, 1 *μ*L of each primer (10 *μ*M), 1 *μ*L LATaq polymerase (Takara; Takara Bio; Mountain View, CA, USA), 25 *μ*L 2x buffer with GC, 3 *μ*L of pure genomic DNA, and nuclease-free water up to 50 *μ*L. The PCR condition for those two-primer pairs was identical. As many as 30 PCR cycles consist of the following steps: initial denaturation at 94°C for 5 minutes, denaturation at 94°C for 2 minutes, annealing at 60°C for 1 minute, extension at 72°C for 1 minute, and final elongation at 72°C for 10 minutes.

## 3. Results and Discussion

### 3.1. Isolation and Screening of Potential Endophytic Bacteria

There are 29 endophytic bacterial isolates successfully isolated from the roots, leaves, and stem of *A. flava* through the serial dilution method. These isolates have different morphological characteristics as mentioned in Table 1. 29 isolates were then roughly screened for antimicrobial activity using the cross-streak method against *S. aureus, E. coli, P. aeruginosa, and C. albicans*. The potential antimicrobial isolates show a clear zone around the colony (Table 1).

**Figure 2:**
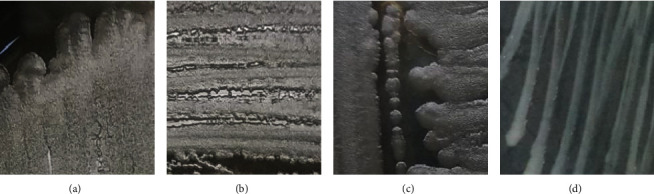
Colony morphology of selected potential endophytic isolates (a) AKEBG21, (b) AKEBG23, (c) AKEBG25, and (d) AKEBG28.

According to the screening results, four potential isolates qualitatively inhibited the growth of *S. aureus* and *E. coli,* namely, AKEBG21, AKEBG23, AKEBG25, and AKEBG28. These all isolates are obtained from the stem of *A.flava* and have similar morphological characteristics such as pale-colored colony, rod-shaped, and Gram-positive(Figure 2). These isolates will be used for the further antimicrobial assay.

### 3.2. Antimicrobial Activity of Selected Endophytic Bacteria

Four selected isolates were tested for further antimicrobial activity against *E. coli*, *P. aeruginosa*, *S. aureus*, and *C. albicans*. The isolates were grown in TSB overnight and centrifuged to concentrate the cells and separate the cells and its medium. The assay was conducted for the pure isolates, pelleted cells, supernatant (medium), and ethyl acetate extract for the isolates. According to the assay, all isolates can inhibit the growth of *E. coli* and *S. aureus* either in the form of pure isolates, pelleted cells, supernatant (medium), and ethyl acetate extract. However, only the ethyl acetate extract of all isolates can inhibit *P. aeruginosa* with relatively higher activity than filtrates and pelleted cells (Table 2).

The isolate AKEBG23 has the highest and most stable antimicrobial activity among the four isolates against the pathogenic bacteria tested. Generally, the antimicrobial activity was found higher in pure isolates and ethyl acetate extract than in pelleted cells and supernatant. Previous research reported that the cell-free supernatant from bacterial isolates mostly had no antimicrobial activity against *S. aureus* and *B. subtilis*. However, the crude extract of those isolates had clear antimicrobial activity [19]. Conversely, Danilova et al. [20] reported that the supernatant of *Lactobacillus plantarum* had antimicrobial activity against food spoilage and pathogenic bacteria. It is allegedly due to the two kinds of antimicrobial compounds produced extracellularly by the bacteria. According to these former research works, it can be known that the antimicrobial compound can be found either in the cell-free supernatant or in the crude extract of the bacteria.

### 3.3. Identification of Selected Potential Isolates

**Figure 3:**
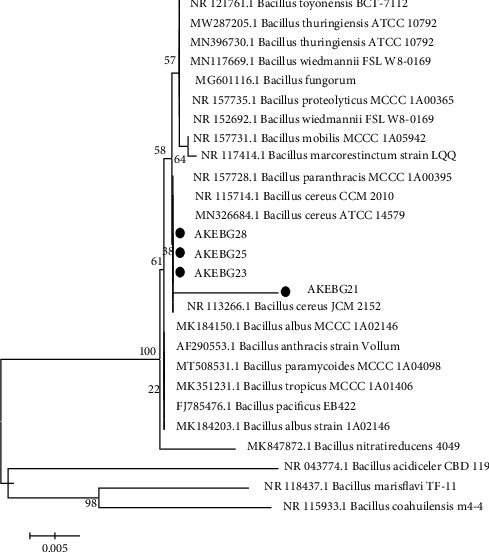
Neighbor-joining tree of four potential bacterial isolates with the highest antimicrobial activity. The tree was tested using the bootstrap method with 1000x replicates.

The four potential isolates obtained were identified through the sequencing of 16S rDNA. The 16S rDNA sequences are compared with the gene bank online database using BLAST-N [16] with a search set restricted to the type material so that the DNA sequences used for identification are well curated and validly identified. According to the phylogenetic tree analysis (Figure 3), all isolates are closely related to *Bacillus cereus*. It is also supported by the morphological characteristics (Table 1), which mentioned that isolates AKEBG21, AKEBG23, AKEBG25, and AKEBG28 are rod-shaped, Gram-positive bacteria which are the main morphological characteristics of *Bacillus*.

### 3.4. Amplification of PKS and NRPS-Encoding Gene

Apart from the identification, detection of polyketide synthase (PKS) and nonribosomal peptide synthase (NRPS)-encoding genes was also conducted to determine the potential of each isolate to produce the bioactive compound. The detection of PKS and NRPS-encoding genes was carried out using the specific primer pair degKS2F.gc/degKSR5.gc for PKS which produce 700 bp amplicon and degNRPS-1F.i/degNRPS-4R.i for NRPS which produces 1000 bp amplicon [18].

According to the amplification, the four selected isolates have PKS and NRPS genes which indicate the potential to produce the bioactive compound. The sequence analysis through BLAST-X B. cereus AKEBG21 PKS gene is closely related to 6-deoxyerythronolide-B synthase, glutamate-1-semialdehyde 2,1-aminomutase from Enterobacter cloacae, and B. cereus AKEBG23, AKEBG25, and AKEBG28 PKS genes are close with Bacillus subtilis ketosynthase type I (Table 3). 6-deoxyerythronolide-B synthase and glutamate-1-semialdehyde 2,1-aminomutase from *B. cereus* AKEBG21 were detected as closely related enzymes from the KS-domain of AKE. According to the conserved domain database, it shares the similar PksD domain with polyketide synthase (PKS) under the superfamily condensing enzyme and decarboxylating condensing enzyme family [21].

The NRPS gene analysis shows that all amplified NRPS fragments were closely related to the *Bacillus* NRPS gene with various similarities (Table 4). Previous research has showed that *Bacillus subtilis* and *Bacillus flexus* with antimicrobial activity against *Vibrio* species were also harboring PKS and NRPS genes [22]. Polyketide synthase (PKS) and nonribosomal peptide synthetase are the enzymes with multidomains which are responsible for bioactive compound production in many microorganisms, specifically bacteria, actinobacteria, and fungi. Moreover, the NRPS gene has a significant role with PKS in bioactive compound biosynthesis which is composed of peptides such as polypeptides and lipopeptides [23].

### 3.5. Profiling of the Active Compound

**Figure 4:**
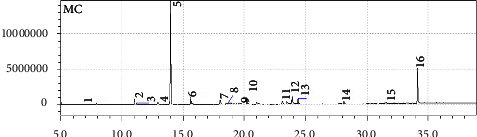
GC-MS profile of ethyl acetate extract from B. cereus AKEBG23.

As the isolate with the highest antimicrobial activity, the GC- MS profile of *B. cereus* AKEBG23 ethyl acetate extract shows that there are 16 bioactive compounds identified in the crude extract (Figure 4). These bioactive compounds were allegedly involved directly and indirectly in the antimicrobial activity of *B. cereus* AKEBG23 against four pathogenic bacteria. There are five major compounds associated with antimicrobial activity, namely, butylated hydroxytoluene, diisooctyl phthalate, E-15-heptadecenal, 1-heneicosanol, and E-14-hexadecenal (Table 5).

Butylated hydroxytoluene (BHT) is known as an antioxidant, which is commonly used as a standard antioxidant [51]. This compound was found as the highest detected compound in GC-MS analysis. Instead of controlling the oxidation process, the bioactivity of BHT is also reported as antimicrobial against *Staphylococcus aureus* [52]. Another research also reported that BHT was also found in the extract of green algae *Scenedesmus obliquus* with antimicrobial bioactivity [31].

The second highest detected compound is diisooctyl phthalate which is mostly known as human-made pollutant phthalic acid esters (PAE). However, several research studies reported that PAE is produced by plants and microorganisms. PAE is also reported to have allelopathic, antimicrobial, and insecticidal activities which increase the competitiveness of the producer [53]. Diisooctyl phthalate is one of the PAE produced by fungi *Fusarium oxysporum* and *Phoma herbarum* and microalgae *Nostoc* sp.; it also has the antimicrobial activity against *Staphylococcus aureus, Klebsiella pneumoniae, Trichophyton mentagrophytes,* and *Candida albicans* [50, 54]. The reports related to diisooctyl phthalate produced by bacteria are limited; this can be a new report that finds diisooctyl phthalate in bacteria, especially *B. cereus.*

Another major compound found in *B. cereus* AKEBG23 ethyl acetate extract is E-15-heptaedecenal, 1-heneicosanol, and E-14-hexadecenal. E-15-heptadecenal and E-14-hexadecenal are reported to be involved in antifungal activity. E-15-heptaedecenal is found in the *B. siamensis* active compound profile which is allegedly involved in antimicrobial activity of *B. siamensis* C.38 against some phytopathogenic fungi [32], while E-14-hexadecenal is the antifungal bioactive compound found in the endophytic fungi *Pseudarthria viscida* (L.) [25, 55]. 1-Heneicosanol is the long-chain fatty alcohol which is reported to have antimicrobial activity against some pathogenic bacteria. Several research studies reported that 1-heneicosanol is found in the plant *Senecio coluhuapiensis* and actinomycetes *Streptomyces carpaticus* [39, 56].

## Figures and Tables

**Table 1:** Morphological characteristics and screening of endophytic bacterial isolates obtained from *A. flava* (L.).

Sources	Isolate code	Gram staining	Cell shape	Inhibition activity			
				*S. aureus*	*E. coli*	*P. aeruginosa*	*C. albicans*
Leave	AKEDT4	Positive	Rod shape	−	−	−	−
	AKEDT5	Negative	Rod shape	−	−	−	−
	AKEDT10	Positive	Rod shape	−	−	−	−
	AKEDT11	Positive	Rod shape	−	−	−	−
	AKEDT12	Positive	Round shape	−	−	−	−

Stem	AKEBT1	Positive	Rod shape	−	−	−	−
	AKEBT2	Positive	Rod shape	−	−	−	−
	AKEBT13	Positive	Round shape	−	−	−	−
	AKEBT14	Positive	Round shape	−	−	−	−
	AKEBT15	Positive	Round shape	−	−	−	−
	AKEBT16	Positive	Round shape	−	−	−	−
	AKEBT17	Positive	Round shape	−	−	−	−
	AKEBT18	Positive	Round shape	−	−	−	−
	AKEBG19	Positive	Rod shape	−	−	−	−
	AKEBG20	Positive	Rod shape	−	−	−	−
	AKEBG21	Positive	Rod shape	+	+	−	−
	AKEBG22	Positive	Rod shape	−	−	−	−
	AKEBG23	Positive	Rod shape	+	+	−	−
	AKEBG24	Positive	Rod shape	−	−	−	−
	AKEBG25	Positive	Rod shape	+	+	−	−
	AKEBG26	Positive	Rod shape	−	−	−	−
	AKEBG27	Positive	Rod shape	−	−	−	−
	AKEBG28	Positive	Rod shape	+	+	−	−
	AKEBG29	Positive	Rod shape	−	−	−	−

Root	AKEAT3	Positive	Rod shape	−	−	−	−
	AKEAT6	Positive	Rod shape	−	−	−	−
	AKEAT7	Positive	Rod shape	−	−	−	−
	AKEAT8	Positive	Rod shape	−	−	−	−
	AKEAT9	Positive	Rod shape	−	−	−	−

**Table 2:** The antimicrobial assay of four selected endophytic isolates.

Sources	Isolate codes	Inhibition zone diameter (mm)			
		Pathogenic microbes			
		*E. coli*	*S. aureus*	*P. aeruginosa*	*C. albicans*
Pure isolates (first screening)	AKEBG21	2.70 ± 0.23	12.7 ± 1.19	0.00 ± 0.00	0.00 ± 0.00
	AKEBG23	22.20 ± 1.20	23.8 ± 0.70	0.00 ± 0.00	0.00 ± 0.00
	AKEBG25	12.70 ± 0.98	19.5 ± 0.45	0.00 ± 0.00	0.00 ± 0.00
	AKEBG28	15.80 ± 1.75	16.7 ± 0.11	0.00 ± 0.00	0.00 ± 0.00
	PC+^*∗*^	20.80 ± 0.22	22.40 ± 0.03	18.30 ± 017	25.08 ± 0.02
	NC−^*∗*^	0.00 ± 0.00	0.00 ± 0.00	0.00 ± 0.00	0.00 ± 0.00

Pelleted cells (centrifuged cells)	AKEBG21	2.10 ± 0.56	1.40 ± 0.56	0.00 ± 0.00	0.00 ± 0.00
	AKEBG23	4.00 ± 0.70	1.00 ± 0.00	0.00 ± 0.00	0.00 ± 0.00
	AKEBG25	1.10 ± 0.07	0.50 ± 0.28	0.00 ± 0.00	0.00 ± 0.00
	AKEBG28	3.10 ± 0.07	0.70 ± 0.28	0.00 ± 0.00	0.00 ± 0.00
	PC+^*∗*^	23.60 ± 0.45	19.50 ± 0.54	18.34 ± 0.23	20.00 ± 0.01
	NC−^*∗*^	0.00 ± 0.00	0.00 ± 0.00	0.00 ± 0.00	0.00 ± 0.00

Medium supernatant	AKEBG21	0.40 ± 0.14	0.15 ± 0.07	0.00 ± 0.00	0.00 ± 0.00
	AKEBG23	3.00 ± 0.28	2.50 ± 0.42	0.00 ± 0.00	0.00 ± 0.00
	AKEBG25	0.95 ± 0.07	1.65 ± 0.07	0.00 ± 0.00	0.00 ± 0.00
	AKEBG28	2.40 ± 0.14	0.50 ± 0.28	0.00 ± 0.00	0.00 ± 0.00
	PC+^*∗*^	23.60 ± 0.45	19.50 ± 0.54	18.34 ± 0.23	20.00 ± 0.02
	NC−^*∗*^	0.00 ± 0.00	0.00 ± 0.00	0.00 ± 0.00	0.00 ± 0.00

Crude extract (ethyl acetate)	AKEBG21	19.10 ± 0.84	13.85 ± 0.49	3.15 ± 1.82	0.00 ± 0.00
	AKEBG23	20.85 ± 0.07	23.15 ± 1.34	22.65 ± 0.21	0.00 ± 0.00
	AKEBG25	19.80 ± 0.50	14.7 ± 0.28	11.65 ± 0.63	0.00 ± 0.00
	AKEBG28	21.50 ± 0.42	14.6 ± 0.00	13.35 ± 0.63	0.00 ± 0.00
	PC+^*∗*^	16.40 ± 0.28	19.5 ± 0.00	18.25 ± 0.63	16.0 ± 0.0
	NC−^*∗∗*^	0.00 ± 0.00	0.00 ± 0.00	0.00 ± 0.00	0.00 ± 0.00

Positive control: streptomycin 1000 ppm for bacteria (*S. aureus, E. coli,* and *P. aeruginosa*; kanamycin 1000 ppm for *C. albicans*. Negative control: NC−^*∗*^: sterile TSB medium and NC−^*∗∗*^: ethyl acetate.

**Table 3:** BLAST-X result of KS-domain amplified from four selected endophytic isolates.

Isolate code	Acc. no.	Description	Similarity (%)	*E*-value
AKEBG21	VAM49526.1	6-deoxyerythronolide-B synthase, Glutamate-1-semialdehyde 2,1-aminomutase (*Enterobacter cloacae*)	99.46	7e-117
	WP_203462309.1	Aminotransferase class III-fold pyridoxal phosphate-dependent enzyme (*Enterobacter cloacae*)	99.46	7e-117
AKEBG23	SAJ35050.1	Ketosynthase (*Bacillus subtilis*)	95.87	2e-152
	WP_032727815.1	Type I polyketide synthase (*Bacillus subtilis*)	95.41	7e-148
AKEBG25	WP_032727815.1	Type I polyketide synthase (*Bacillus subtilis*)	98.97	8e-137
	WP_187956203.1	Polyketide synthase PksL (*Bacillus subtilis*)	98.97	5e-128
AKEBG28	SAJ35050.1	Ketosynthase (*Bacillus subtilis*)	99.53	2e-159
	WP_032727815.1	Type I polyketide synthase (*Bacillus subtilis*)	99.07	8e-155

**Table 4:** BLAST-X result of NRPS domain amplified from four selected endophytic isolates.

Isolate codes	Acc. no.	Description	Similarity (%)	*E*-value
AKEBG21	WP_098675317.1	Nonribosomal peptide synthetase (*Bacillus thuringiensis*)	99.67	0.00
	WP_098276241.1	Nonribosomal peptide synthetase, partial (*Bacillus thuringiensis*)	99.34	0.00
AKEBG23	WP_000503024.1	MULTISPECIES: nonribosomal peptide synthetase (*Bacillus*)	99.29	0.00
	WP_088053951.1	Nonribosomal peptide synthetase (*Bacillus cereus*)	99.29	0.00
AKEBG25	WP_000503024.1	MULTISPECIES: nonribosomal peptide synthetase (*Bacillus*)	99.67	0.00
	WP_059303799.1	Nonribosomal peptide synthetase (*Bacillus cereus*)	99.67	0.00
AKEBG28	WP_000503030.1	Nonribosomal peptide synthetase (*Bacillus thuringiensis*)	80.27	0.00
	WP_088053951.1	Nonribosomal peptide synthetase (*Bacillus cereus*)	80.27	0.00

**Table 5:** Bioactive compound profile of *B. cereus* AKEBG23.

No	Retention time	% Area	Name	Chemical formula	Similarity (%)	Bioactivity
1	7.908	0.39	1-Dodecanol	C_12_H_26_O	95	Antibacterial activity [24]
2	11.027	2.20	E-14-hexadecenal	C_16_H_30_O	95	Antifungi [25]
3	11.172	0.48	Tetradecane	C_14_H_30_	95	Antioxidant, antimicrobial [26], and anti-inflammatory [27]
4	12.951	0.78	Cyclodeca[b]furan-2(3H)-one, 3a,4,5,6,7,8,9,11a-octahydro-3,6,10-trimethyl-	C_15_H_24_O_2_	75	Antioxidant activity [28]
5	13.986	57.13	Butylated hydroxytoluene	C_15_H_24_O	95	Antioxidant [29, 30]; antimicrobe [31]
6	15.637	3.55	E-15-heptadecenal	C_17_H_32_O	96	Antimicrobe [32, 33]
7	18.034	3.04	2-Propenoic acid, tetradecyl ester	C_17_H_32_O_2_	88	—
8	18.716	0.87	Spiro-1-(cyclohex-2-ene)-2′-(5′-oxabicyclo(2.1.0)pentane), 1′,4′,2,6,6-pentamethyl-	C_14_H_22_O	76	Biomarkers to detect the presence of toxigenic fungal pathogen [34]
9	19.574	0.58	Phenol, 4-(1,1,3,3-tetramethylbutyl)-	C_14_H_22_O	92	—
10	20.220	3.06	E-15-heptadecenal	C_17_H_32_O	95	Antimicrobe [35]
11	23.083	2.57	Pyrrolo (1,2-a) pyrazine-1,4-dione, hexahydro-3-(2-methylpropyl)-	C_11_H_18_N_2_O_2_	90	Antioxidant [36]
12	23.885	5.06	l-(+)-Ascorbic acid 2,6-dihexadecanoate	C_38_H_68_O_8_	86	Antimicrobial and antitumor [37, 38]
13	24.377	2.26	1-Heneicosanol	C_21_H_44_O	95	Antimicrobe and antifungi [39]; Antimycobacteria [40, 41]
14	28.133	1.00	n-Tetracosanol-1	C_24_H_50_O	94	Antibacterial [42]; anti-inflammatory [43]; antioxidant [44]
15	31.556	0.42	1-Heptacosanol	C_24_H_38_O_4_	93	Antibacterial [45]; antifungal [46]
16	34.145	16.61	*Diisooctyl phthalate*	C_24_H_38_O_4_	96	Antimicrobe [47–49]; antitumor [50]

## Data Availability

The GC-MS, PKS, and NRPS sequence data used to support the findings of this study are available from the corresponding author upon request.
